# Supplementary material for: Donor transcription suppresses D-loops in cis and promotes genome stability
Source: EMBO J. 2025 Aug 26;44(19):5595–617. doi: 10.1038/s44318-025-00541-x (PMC12489061; doi:10.1038/s44318-025-00541-x)
Supplement: Supplementary file 5 — Dataset EV1 [file 44318_2025_541_MOESM5_ESM.zip › Dataset EV1/README.rtf]

Dataset EV1: Annotated sequences of the genetic constructs used in this study
Sequences are annotated in a Genbank format. 
Donor sequences show promoter, homology region and terminator sequences highlighted. File name indicates whether the donor is at the lys2, can1 or ade2 locus.
DSB-inducible sequences at ura3 show homology region and HOcs position. 
Other sequences show the empty or RNH1-containing over-expression pYES2 vector.
