# Supplementary material for: Donor transcription suppresses D-loops in cis and promotes genome stability
Source: EMBO J. 2025 Aug 26;44(19):5595–617. doi: 10.1038/s44318-025-00541-x (PMC12489061; doi:10.1038/s44318-025-00541-x)
Supplement: Supplementary file 13 — Expanded View Figures [file 44318_2025_541_MOESM13_ESM.pdf]

Expanded View Figures

Figure EV1. Co-directional donor transcription suppresses nascent D-loops (related to Fig. 1).

(**A**) Rationale of the proximity ligation-based D-loop Capture (DLC) and D-loop Extension (DLE) assays. (**B**) ChIP-qPCR of the Rpb1 subunit of RNA Pol II at the 5′ and 3′ end of the donor 2 h post-DSB induction. (**C**) RT-qPCR of the RNA produced at the donor and at the downstream *RAD16* gene 2 h post-DSB induction. (**D**) Multiple controls of the DLC experiments with varying transcriptional levels at the donor site. Left: Quantification of the DSB frequency at *HOcs* 2 h post-DSB induction. Middle: control of the EcoRI digestion efficiency in the broken and donor molecules. Right: Circularization efficiency of a *GAL3*-containing fragment as a function of its transcriptional activity. (**E**) Correlation between the D-loop levels and the RNA Pol II amount at the donor (left) and donor transcript (right). Data show mean ± SEM. From data in (**B**) (Pol II IP), (**C**) (RT-qPCR), and Fig. 1C (DLC). (**F**) RT-qPCR of the RNA produced at the 1-kb- and 2-kb-long donors 2 h post-DSB induction. (**B–D**, **F**) Data points show individual biological replicates (*n*). Bars show mean ± SEM.

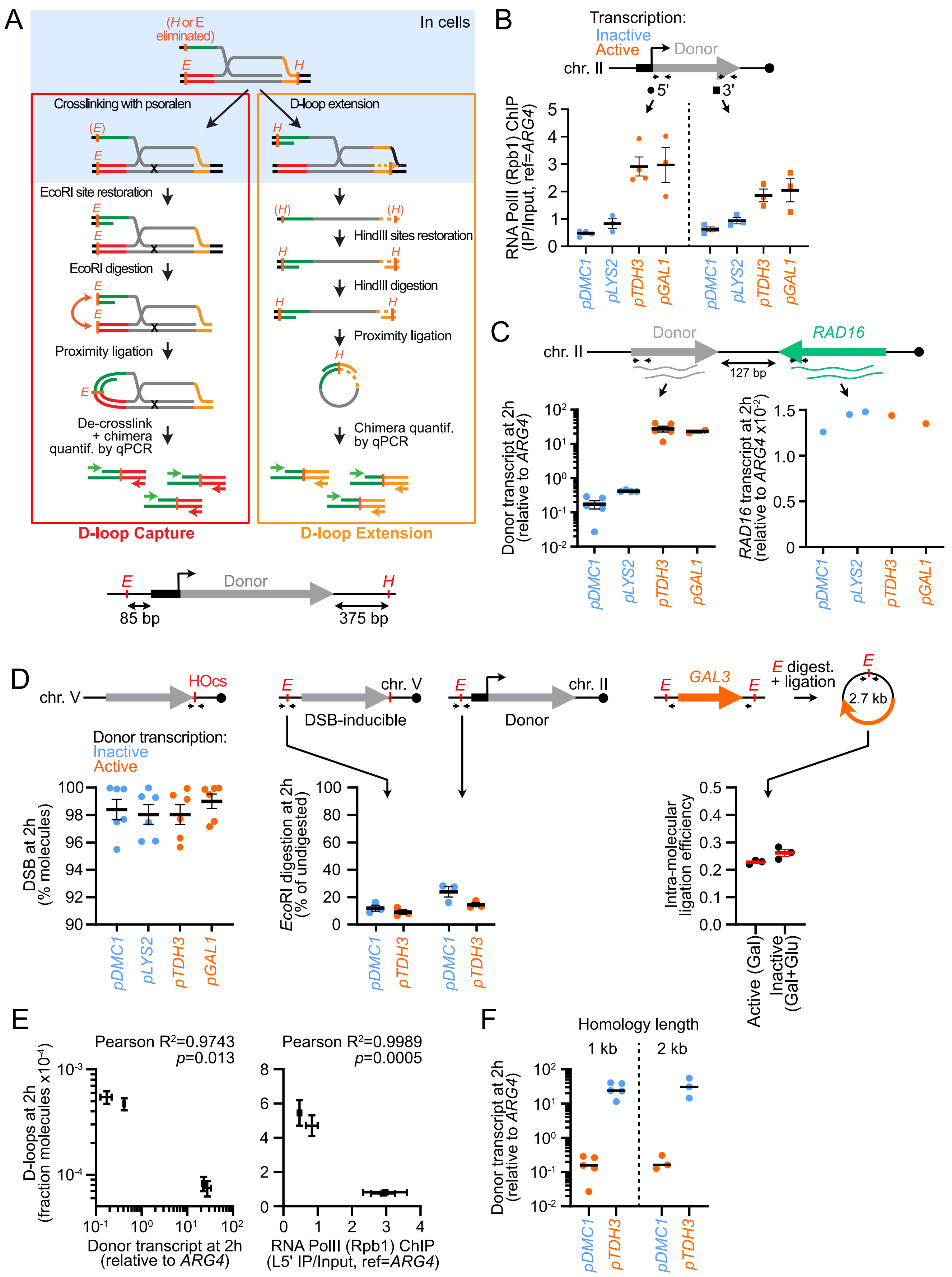
A
In cells
(H or E eliminated)
Crosslinking with psoralen
D-loop extension
EcoRI site restoration
EcoRI digestion
Proximity ligation
De-crosslink + chimera quantif. by qPCR
HindIII sites restoration
HindIII digestion
Proximity ligation
Chimera quantif. by qPCR
D-loop Capture
D-loop Extension
Donor
85 bp
375 bp
B
Transcription:
Inactive
Active
Donor
chr. II
5'
3'
RNA PolII (Rpb1) ChIP (IP/Input, ref=ARG4)
pDMC1
pLYS2
pTDH3
pGAL1
C
chr. II
Donor
RAD16
127 bp
Donor transcript at 2h (relative to ARG4)
RAD16 transcript at 2h (relative to ARG4 x10⁻²)
D
chr. V
HOcs
chr. V
DSB-inducible
chr. II
Donor
GAL3
E digest. + ligation
2.7 kb
Donor transcription:
Inactive
Active
DSB at 2h (% molecules)
EcoRI digestion at 2h (% of undigested)
Intra-molecular ligation efficiency
Active (Gal)
Inactive (Gal+Glu)
E
Pearson R²=0.9743
p=0.013
Pearson R²=0.9989
p=0.0005
D-loops at 2h (fraction molecules x10⁻⁴)
Donor transcript at 2h (relative to ARG4)
RNA PolII (Rpb1) ChIP (L5' IP/Input, ref=ARG4)
F
Homology length
1 kb
2 kb
Donor transcript at 2h (relative to ARG4)

© The Author(s)

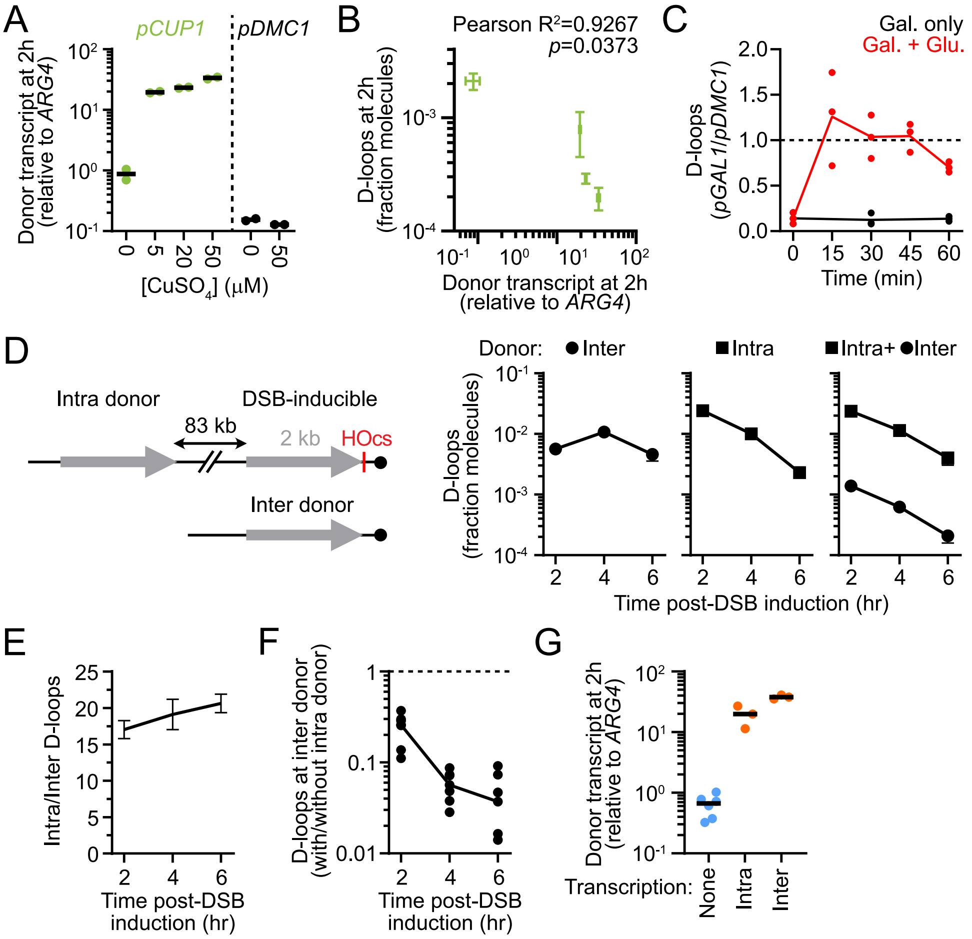

Figure EV2. Donor transcription suppresses D-loops in *cis* (related to Fig. 2).

(**A**) RT-qPCR of the RNA produced at the donor with varying copper concentrations 2 h post-DSB induction. (**B**) Correlation between the D-loop levels and donor transcript levels with varying copper concentrations. Data show mean ± SEM. From data in (**A**) (RT-qPCR) and Fig. 2A (DLC). (**C**) Ratio of D-loops formed at the donor site under the control of the *pGAL1* over the *pDMC1* promoter in contexts in which the *pGAL1* promoter is active (Gal. only) or at increasing time post-shutoff (Gal. + Glu.). From data in Fig. 2B. (**D**) Kinetics of D-loop levels at an intra-chromosomal and/or an inter-chromosomal donor (APY266, APY826, and APY809). Data show mean ± SEM of $n \geq 7$ (inter donor only), $n = 1$ (intra donor only), and $n \geq 7$ (intra+inter donor) biological replicates. (**E**) Ratio of D-loops formed at the intra donor over the inter donor when both are present (APY809). From data in (**C**). Data show mean ± SEM of $n \geq 7$. (**F**) Fold decrease of the D-loop at the inter donor when the intra donor is present. From data in (**D**) acquired in parallel ($n = 6$). (**G**) RT-qPCR of the donor RNA produced in strains bearing an intra and an inter donor either non-transcribed (APY809), with the intra donor transcribed (APY1587), or with the inter donor transcribed (APY1709). (**A**, **C**, **F**, **G**) Data points show individual biological replicates (*n*). Bars show mean ± SEM.

© The Author(s)

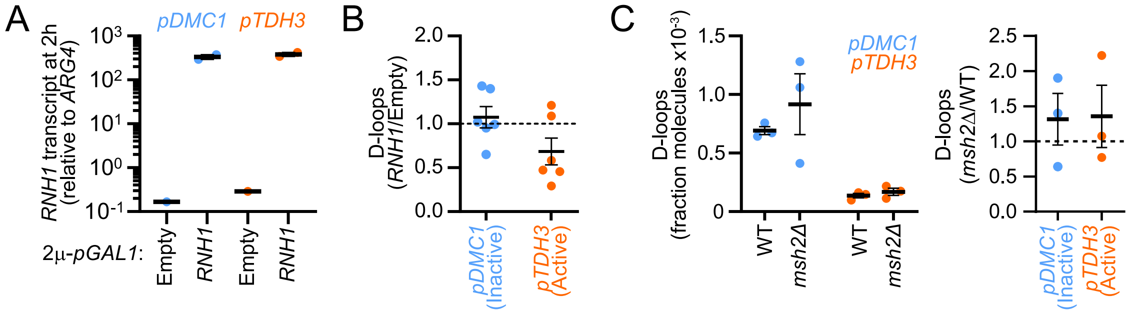

Figure EV3. Genetic determinants of transcription-mediated D-loop suppression (related to Fig. 3).

(**A**) *RNH1* transcript levels determined by RT-qPCR as a function of the overexpression vector 2 h post-DSB induction. (**B**) D-loops level fold change at transcriptionally inactive and active donors upon *RNaseH1* overexpression. From data in Fig. 3A. (**C**) Left: D-loop levels at transcriptionally inactive and active donors in a WT (APY502 and APY725) and a *msh2Δ* (APY1608 and APY1610) strain. Right: relative mutant values compared to a WT strain assayed in parallel. No statistically significant differences were detected between *msh2Δ* and WT cells. (**A–C**) Data points show individual biological replicates (*n*). Bars show mean ± SEM.

© The Author(s)

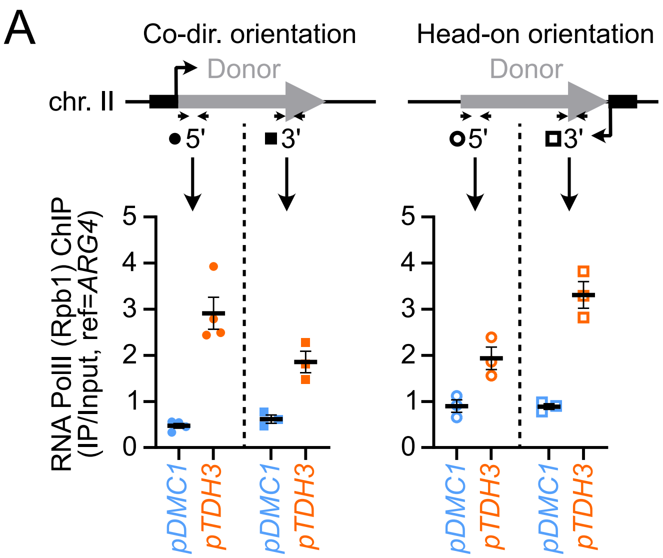

Figure EV4. Effect of transcription directionality on RNA Pol II enrichment at the donor (related to Fig. 4).

(**A**) ChIP-qPCR of the Rpb1 subunit of RNA Pol II at the 5′ and 3′ end of the donor 2 h post-DSB induction with promoters in the co-directional or head-on orientation.

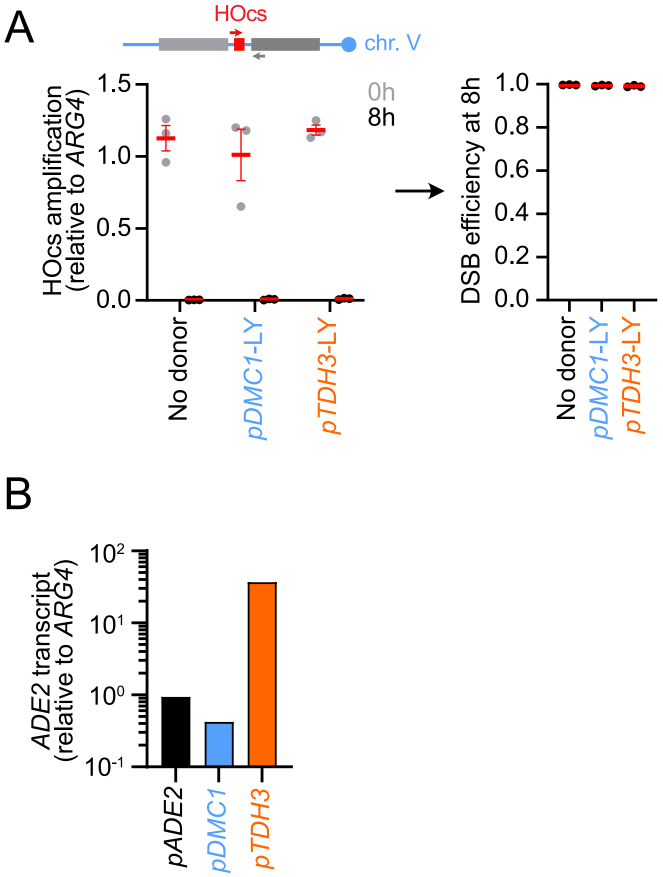

Figure EV5. Controls for the ectopic and allelic recombination systems.

(**A**) Efficiency of DSB formation in the ectopic repair system. Left: Amplification across the HOcs prior to and 8 h post-DSB induction. Right: Deduced DSB efficiency at 8 h post-DSB induction. Data points show individual biological replicates (*n*). Bars show mean ± SEM. (**B**) Abundance of the *ADE2* transcript in diploid cells bearing the *ade2-n* donor under control of different promoters, scored after 2 h of DSB induction and 1 h of induction shutoff upon glucose addition in liquid media. This condition best matches the context in which DSB repair is expected to take place upon plating cells on YPD media following I-SceI induction for 2 h in liquid media. Data shows a single biological replicate ($n = 1$).

 © The Author(s)

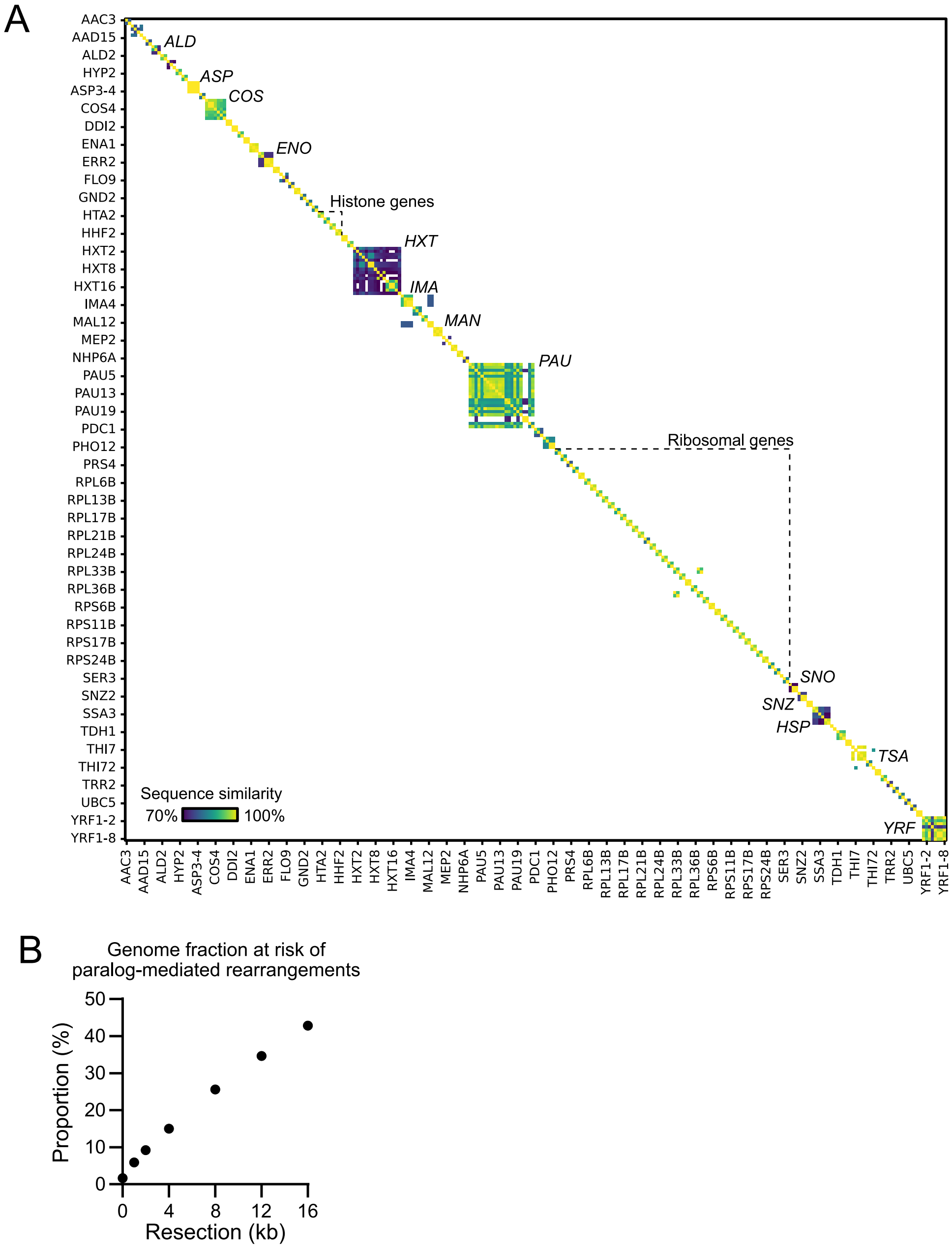

Figure EV6. Analysis of the DNA sequence similarity of paralogous genes and their potential impact on genome stability (related to Fig. 6).

(**A**) Pairwise similarity matrix for paralogous genes exhibiting >70% sequence similarity, obtained from Dataset EV2. (**B**) Fraction of the genome exposing >300 bp of at least one paralogous gene in (**A**) as a function of the resection length at randomly distributed DSBs.
